# The impact of lipid-rich nutrition on ketogenesis and muscle weakness in sepsis

**DOI:** 10.1186/s40635-026-00867-8

**Published:** 2026-02-12

**Authors:** Caroline Lauwers, Jan Gunst, Soraya El Dawy, Sarah Derde, Lies Pauwels, Inge Derese, Sarah Vander Perre, Greet Van den Berghe, Michael P. Casaer, Lies Langouche

**Affiliations:** Department of Cellular and Molecular Medicine, Laboratory and Clinical Division of Intensive Care Medicine, KU Leuven, Herestraat 49, Box 503, 3000 Leuven, Belgium

**Keywords:** Sepsis, Critical illness, Muscle weakness, Ketones, Medium-chain triglycerides, Long-chain triglycerides, Carnitine, Ketogenic diet

## Abstract

**Background:**

Administration of ketone bodies attenuated the severity of sepsis-induced muscle weakness in preclinical studies. Whether lipid-rich emulsions may likewise mitigate such muscle weakness by stimulating the endogenous ketogenic capacity remains uncertain, especially in relation to glucose, a critical suppressor of ketogenesis. This study investigated the ketogenic potential of parenteral nutrition rich in long- and/or medium-chain triglycerides with differing glucose content on sepsis-induced muscle weakness.

**Methods:**

We used a parenterally fed murine model of prolonged sepsis-induced muscle weakness to investigate specific lipid mixtures in two consecutive studies. Septic mice receiving standard total parenteral nutrition (TPN) and healthy control (HC) animals were included as references in both studies. In a first study, septic mice received pure long-chain triglycerides (LCT) or long-chain triglycerides supplemented with glucose (gLCT). The second study compared a gLCT mixture to a mixed medium- and long-chain triglyceride emulsion supplemented with glucose (gMCT). After 5 days of sepsis, markers of ketone body metabolism, muscle function, and muscle and liver metabolomics were measured.

**Results:**

In study one, ketosis was undetectable with TPN-treatment, but substantially increased with pure LCT (median 1.39 mmol/L, *p* < 0.001). Supplemental glucose suppressed ketosis sixfold (median 0.24 mmol/L, *p* < 0.001). The sepsis-induced muscle weakness was exacerbated in LCT mice, while muscle force was comparable between TPN-treated and gLCT mice (TPN 60.9%; gLCT 60.9%; LCT 33.1% of HC 128.7 mN/mm^2^, *p* < 0.001). The decrease in muscle glycolytic metabolites in LCT mice relative to TPN-treated mice was attenuated by supplemental glucose. In study 2, unexpectedly, ketosis was similarly low in gLCT and gMCT mice (*p* = 0.1), and muscle force was equally reduced in all septic groups (TPN 68.1%; gLCT 74.0%; gMCT 65.9% of HC 105.9 mN/mm^2^, *p* = 0.5) as compared to HC mice. Protein expression of the rate-limiting enzyme of ketogenesis, Hmgcs2, was suppressed in gMCT as compared to gLCT mice (*p* = 0.04).

**Conclusions:**

Pure LCT infusion induced ketosis, but aggravated muscle weakness, which was attenuated by providing supplemental glucose. Combined with glucose, neither long-chain triglycerides nor mixed medium- and long-chain triglycerides were able to induce adequate ketosis or attenuate sepsis-induced muscle weakness.

**Supplementary Information:**

The online version contains supplementary material available at 10.1186/s40635-026-00867-8.

## Background

Intensive care unit-acquired weakness (ICUAW) has been associated with excess mortality and impaired quality of life [1]. Preventive strategies may attenuate its severity [2]. Among these, relative macronutrient restriction in the first week in ICU lowered the incidence of ICUAW and enhanced its recovery [3]. These beneficial effects were at least partly explained by the induction of a beneficial fasting response, of which ketone bodies are considered a hallmark. Nutritional strategies promoting ketogenesis in critical illness have yet to be systemically investigated.

Ketones are unique metabolites that are produced during low-carbohydrate conditions, serving as an alternative energy protein-sparing substrate, but also activating signaling pathways and thereby promoting resilience to cellular stress and enhancing recovery [4, 5]. Previous animal studies have shown that infusion of ketone bodies increased muscle force during concomitant total parenteral nutrition (TPN) infusion in a model of prolonged murine sepsis [6, 7]. Ketogenic diets, however, are only rarely implemented in the ICU, often for specific reasons such as refractory epilepsy and inborn errors of metabolism. Furthermore, critical illness results in marked alterations in the neuroendocrine axes and metabolic pathways, which may affect the hepatic ketogenic capacity [8]. Indeed, crucial regulators of lipid transport and oxidation and ketogenesis, including the peroxisome proliferator-activated receptor α (PPARα) and carnitine homeostasis, appear suppressed or deficient during critical illness [9–12]. Importantly, these processes may be affected by triglyceride chain-length: long-chain triglycerides (LCTs) are essential lipids, but require carnitine for intramitochondrial transport unlike medium-chain triglycerides (MCTs) [13–15]. Interestingly, a previous animal study unexpectedly revealed an exacerbation in sepsis-induced muscle weakness during pure LCT infusion with pharmacological PPARα activation [16], but the impact of supplemental glucose during LCT infusion was not investigated. The current study therefore aims to investigate the ketogenic potential of TPN either rich in LCT or MCT in combination with glucose and its impact on sepsis-induced muscle weakness in 2 consecutive animal studies.

## Methods

### Animal study design

The animal experiments were approved by the local ethical committee (CMM-077/2023). C57Bl/6JRj male mice (Janvier, Le Genest-Saint-Isle, France), aged 24 weeks, received a central venous catheter in the jugular vein after which polymicrobial abdominal sepsis was induced by a caecal ligation and puncture (CLP) with a 18G needle [17]. Septic mice were fluid resuscitated (Plasmalyte, Baxter^®^, containing 4.2% glucose). Parenteral nutrition (PN) was initiated only after 20 h mimicking the clinical standard practice of withholding PN in the early stages of critical illness. To provide reference values of the standard-of-care nutritional management, a group of septic mice receiving standard total parenteral nutrition (TPN, Olimel N7E, Baxter^®^, provided at an infusion rate of 0.3 ml/h). Ad libidum fed healthy control mice were included to provide reference values. After the termination of the experiment on the 5th day, ex vivo muscle force was measured as the primary outcome. Experiments were completed when muscle force measurement was successfully measured in at least 15 surviving animals. This sample size allows to detect, with > 80% power and > 95% certainty, a mean difference of 34 mN/mm^2^ in specific muscle force between groups, assuming a common standard deviation of 32 [6]. Throughout the experiment, septic mice received pain medication and antibiotic treatment twice daily to comply with current clinical practice, and pain and discomfort was monitored by the Mouse Grimace Pain Score [17, 18]. Additional details of the experimental setup can be found elsewhere [17].

In the first study, the aim was to assess the metabolic interaction between glucose and LCTs in relation to ketogenesis and muscle function. Accordingly, the first intervention group of septic mice received either standard TPN (TPN), a pure LCT emulsion (ClinOleic 20%, Baxter^®^) without any other macronutrients (LCT), or an LCT emulsion with supplemental glucose (gLCT) (mixture of ClinOleic 20%, Baxter^®^ emulsion and 50% glucose, Baxter^®^; of which 10% of the caloric intake comprised of glucose) (Supplementary Table S1 and Figure S1). LCT emulsions were the preferred lipid substrate as they are commonly present in current nutritional regimens in daily clinical practice and prevent the development of essential fatty acid deficiency [13, 19]. Animals were randomly allocated to the different nutritional regimens. All animals were sacrificed by cardiac puncture after the 5-day study period, and plasma and tissue samples were collected right after and stored in −80 °C.

In the second study, we assessed the ketogenic potential of an MCT-enriched vs. a pure LCT emulsion, both supplemented with glucose. Analogous to the first study, septic mice were randomly allocated to either standard TPN (TPN), a pure LCT emulsion supplemented with glucose (gLCT, composition similar as in study 1) or an MCT-enriched emulsion (gMCT) (mixture of Lipofundin 20%, B. Braun^®^, composed of 50% LCT and 50% MCT, and 50% glucose, Baxter^®^; of which 10% of the caloric intake comprised of glucose) (Supplementary Table S1 and Figure S1). All glucose and lipid mixtures aimed to deliver a similar caloric intake as the TPN solution, providing theoretically 5.27 kilocalories per day. All animals were sacrificed by cardiac puncture after a 5-day study period and plasma and tissue samples were collected.

### Ex vivo muscle force measurements

Ex vivo muscle force was measured in all surviving septic mice and healthy control mice in the hindlimb m. extensor digitorum longus (EDL) as the primary outcome. Directly after terminating the experiment, the EDL was measured ex vivo suspended in a temperature controlled organ bath (300C-LR Dual-Mode muscle lever, Aurora Scientific, Ontario, Canada). Maximal isometric tetanic force was measured as the average force generated by three consecutive tetanic stimuli of 180 Hz and divided by the muscle cross-sectional area to calculate the specific maximal isometric tetanic force. More details are provided in the supplemental file.

### Whole blood and plasma analyses

A point-of-care tester (StatStrip Xpress 2; Nova Biomedical, Waltham, MA) measured 3-hydroxybutyrate (3HB) concentrations on whole tail vein blood in all animals. All other measurements were performed on plasma collected with a cardiac puncture at the end (day 5) of the experiment of all surviving animals. Plasma 3HB concentrations were quantified with an internally developed enzymatic assay with a detection limit of 0.005 mmol/L [20]. Commercial assays were used for plasma triglycerides (TG, Abcam, Cambridge, UK), tumor necrosis factor and Il-6 (R&D Systems, Abingdon, UK), and malondialdehyde (MDA, Abcam, Cambridge, UK). More details are provided in the supplemental file.

### Tissue analyses

Water content, gene expression and metabolite tissue content were assessed on the gastrocnemius muscle and liver tissue of all surviving animals. Dry mass was measured by freeze-drying. Relative gene expression was determined by commercial TaqMan^®^ assays (Applied Biosystems, Carlsbad, CA, USA) (Supplementary Table S2). Liver triglycerides were measured (FUJIFILM Wako, Richmond, USA) after hexane extraction. Commercial assays (Abcam, Cambridge, UK) were used to measure muscle glycogen content. Hmgcs2 expression in liver tissue was determined with western blotting. Liver paraffin sections were scored semi-quantitatively for inflammation, necrosis, and steatosis. Muscle sections were assessed semi-quantitatively for inflammation, necrosis, fibrosis and fiber shape. Targeted metabolomics of muscle (first study) and liver (first and second study) tissue were performed with a Dionex UltiMate 3000 LC System (Thermo Scientific Bremen, Germany) equipped with a C-18 column (Acquity UPLC -HSS T3 1. 8 µm; 2.1 × 150 mm, Waters) coupled to a Q Exactive Orbitrap mass spectrometer (Thermo Scientific) by the metabolomics expertise center (KU Leuven). Data analysis of metabolomics data was conducted by the MetaboAnalystR package [21]. Plasma and liver acylcarnitine profile and free carnitine concentrations were assessed by liquid chromatography with tandem mass spectrometry (LC–MS-MS) by the Metabolomics Innovation Centre (Victoria, Canada). More details are provided in the supplemental file.

### Statistics

Median and interquartile ranges were used as data summary measures and represented by boxplots of which the whiskers extend until the furthest point within 1.5 times the interquartile range. After visual inspection of the distribution of the data, the Analysis of Variance (ANOVA) or Student’s t-test were applied in case of a Gaussian distribution, or the Kruskal–Wallis test or Wilcoxon signed-rank test if the data was skewed, as appropriate. Kaplan–Meier survival curves were assessed by the log-rank test. Two-sided p ≤ 0.05 were considered statistically significant. Statistical analyses were conducted with the R statistical programming language (R Core Team (2023). _R: A Language and Environment for Statistical Computing_. R Foundation for Statistical Computing, Vienna, Austria. < https://www.R-project.org/ > ., (version 4.5.0)) and the RStudio interface (version 2025.05.0, Boston, MA, USA).

## Results

### Supplemental glucose infusion suppressed ketosis but reversed the aggravation in muscle weakness during pure LCT infusion

**Fig. 1:**
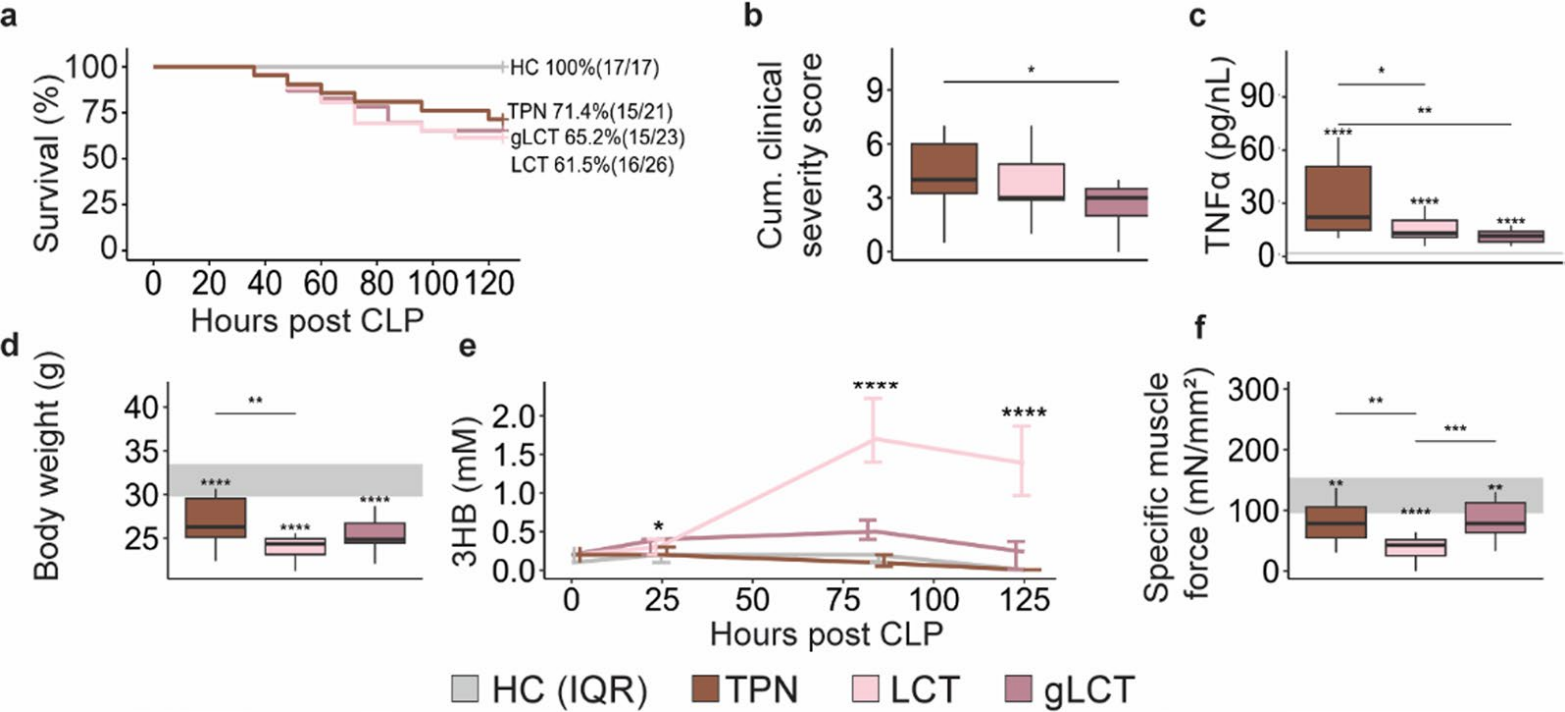
impact of glucose supplemented LCT emulsion vs. a pure LCT emulsion on survival after 5 days of sepsis (**a**), severity of illness scores of surviving animals (**b**), plasma TNFα (**c**) total body weight (**d**), and ketosis (**e**), and specific muscle force (**f**). From the 87 animals included in this experiment, 63 animals survived until the end of the study period: healthy control group n = 17/17; sepsis TPN group n = 15/21; sepsis LCT group n = 16/26; sepsis gLCT group n = 15/23. *CLP* cecal ligation and puncture, *cum.* Cumulative, *TNFα* Tumor Necrosis Factor alpha, *3HB* 3-hydroxybutyrate. The interquartile range of HC mice is shown in gray and asterisks above boxplots denote comparisons with HC mice. Statistical significance is shown by */**/***/****: *p* < 0.05/0.01/0.001/0.0001. In the line plot, overall statistical difference was assessed by the Kruskal–Wallis test for each time point (0 h, 25 h, 84 h and 125 h)

After 5 days of sepsis, survival was comparable among all interventional groups (*p* = 0.8) (Fig. 1a). Severity of illness scores were the lowest in the gLCT group, and plasma TNFα concentrations were lower in the LCT and gLCT groups relative to the TPN group (Fig. 1b, c). Total body weight was lower in LCT mice than TPN mice (Fig. 1d). Hepatic (Il6 and Tnf) and muscle (Il6, Tnf, Il1b and Nlrp3) gene expression markers of inflammation were not different among septic groups (Figure S2). Plasma 3HB concentrations were mostly below the detection limit (0.005 mM) during TPN infusion, while LCT infusion induced a stable ketosis throughout the experiment, reaching a median of 1.4 mM after 5 days of sepsis (Fig. 1e). Supplemental glucose significantly suppressed ketosis, resulting in plasma 3HB concentrations that were almost 6 times lower on the fifth day (median of 0.24 mM) (Fig. 1e). Despite a steady ketosis in the LCT group, specific muscle force declined further in this group as compared to the standard-of-care TPN-treated group (Fig. 1f). Supplemental glucose could reverse this exacerbation in muscle force, but only up to the levels of the TPN-group, in spite of the modest ketosis (Fig. 1f). On histological analysis, signs of necrosis, inflammation, fibrosis and fiber size were overall comparable among the septic mice as were muscle dry weight and water content, and gene expression levels of muscle heavy chains (Figure S3).

### Supplemental glucose during LCT infusion restored impaired glucose metabolism and TCA intermediates during pure LCT infusion

**Fig. 2:**
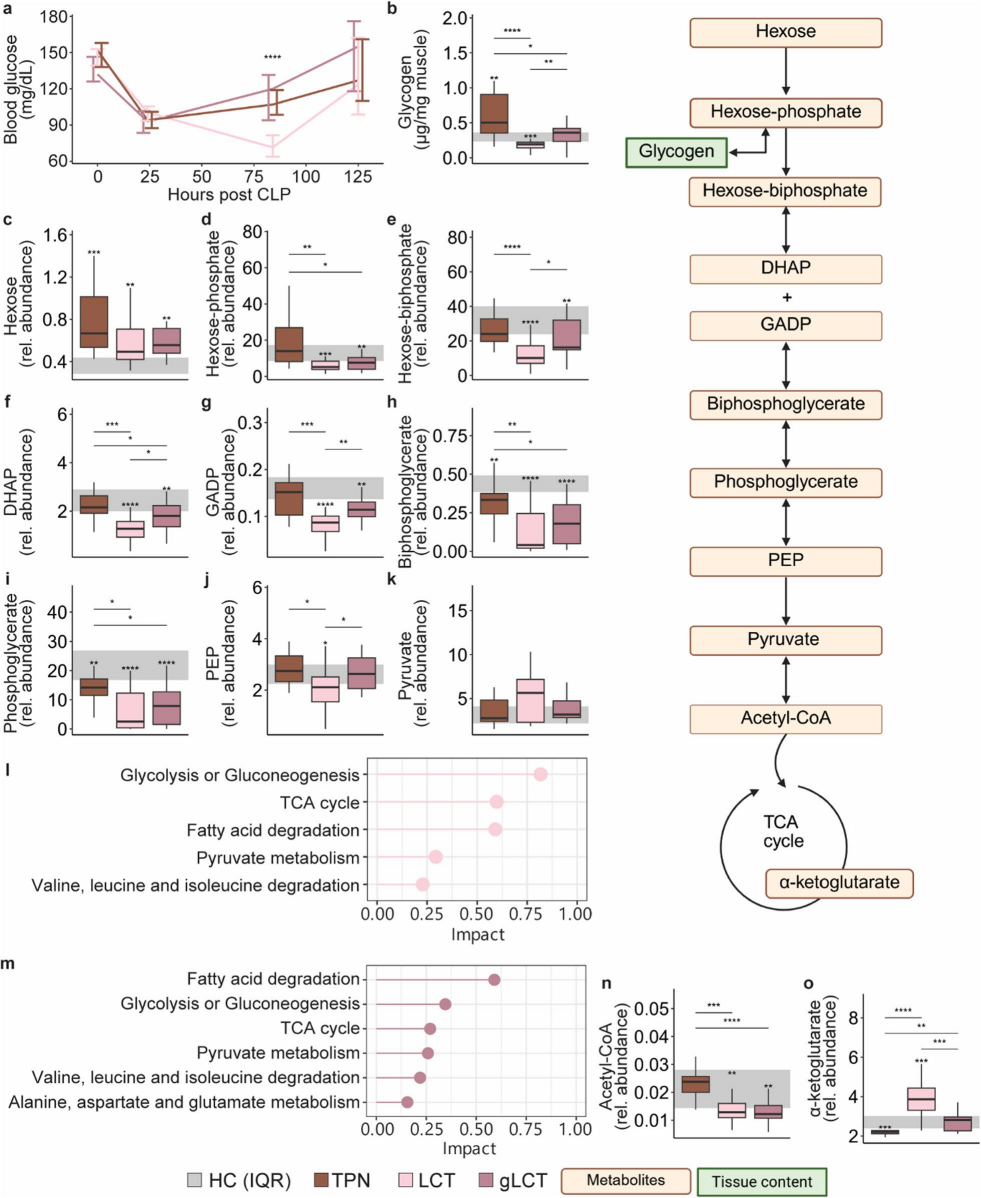
impact of glucose supplemented LCT emulsion vs. a pure LCT emulsion on glycemia (**a**), muscle glycogen content (**b**), glycolytic intermediates (**c**–**j**) and pyruvate (**k**). Joint pathway analysis of muscle metabolomics in the LCT group (**l**) and gLCT group (**m**) relative to the TPN-treated mice. The impact on muscle acetyl-CoA (**n**), and α-ketoglutyrate (**o**). *DHAP* Dihydroxy-acetone phosphate, *GADP* Glyceraldehyde 3-phosphate, *PEP* Phosphoenolpyruvate, *TCA* tricarboxylic acid, *CoA* coenzyme A. The interquartile range of HC mice is shown in gray and asterisks above boxplots denote comparisons with HC mice. Statistical significance is shown by */**/***/****: p < 0.05/0.01/0.001/0.0001. Illustrations were created with BioRender.com

To comprehend the mechanism by which pure LCT infusion exacerbated sepsis-induced muscle weakness and the additional glucose load reversed this phenotype, metabolic pathways in muscle tissue were further explored. Blood glucose concentrations were lower in the LCT group only on the third day of the experiment compared to the other septic mice (Fig. 2a). As compared to TPN-treated septic mice and HC mice, muscle glycogen stores and glycolytic intermediates were decreased in the LCT group (Fig. 2b–j). This was supported by joint pathway analysis, which identified glucose-related pathways as substantially altered in this group relative to the TPN-treated mice (Fig. 2l). This decline in muscle glycogen and glucose-related metabolites was less pronounced when supplemental glucose was provided (Fig. 2b–j). Muscle pyruvate levels were comparable among the septic groups, but acetyl-CoA levels were similarly reduced in both lipid groups compared to the TPN group (Fig. 2k, n). Only within the LCT group, pyruvate and acetyl-CoA had a strong negative correlation (Spearman correlation = −0.71, *p* = 0.003, other groups: Spearman correlation <|0.37| and *p* > 0.05). Most TCA metabolites decreased during TPN administration as compared to HC mice (Fig. 2o and Figure S4). LCT infusion increased most TCA intermediates, in particular α-ketoglutarate, which was markedly elevated in this group as compared to the other groups (Fig. 2o). The addition of supplemental glucose to the LCT infusion normalized TCA intermediates including α-ketoglutarate Fig. 2o and Figure S4).

### Supplemental glucose during LCT infusion did not enhance muscle lipid and ketone body metabolism

**Fig. 3:**
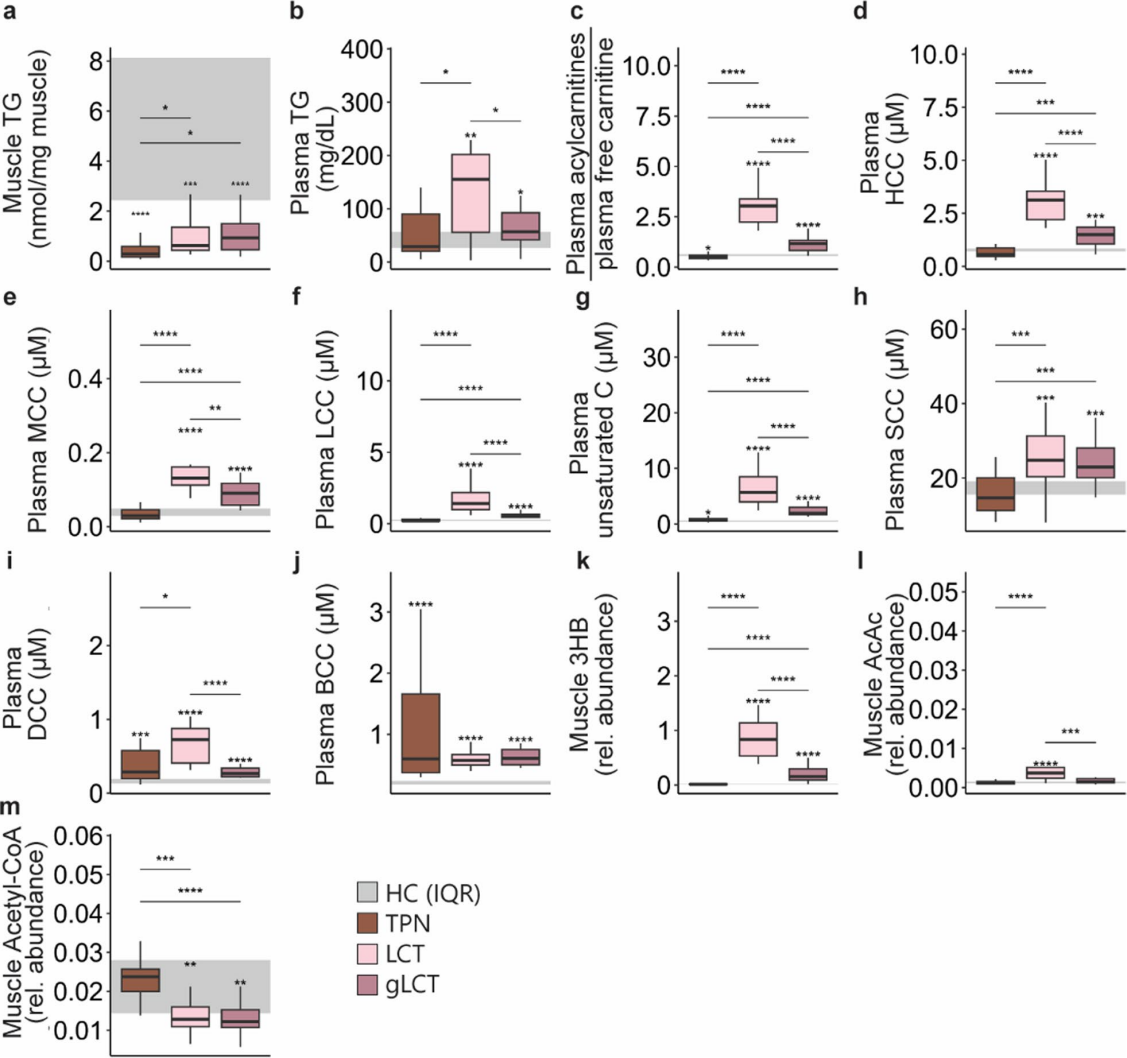
impact of a glucose supplemented LCT emulsion vs. a pure LCT emulsion on muscle (**a**) and plasma TG (**b**), the ratio of plasma acylcarnitines to free carnitine (**c**), plasma hydroxylated (**d**), medium-chain (**e**), long-chain (**f**), unsaturated (**g**), short-chain (**h**), dicarboxylic (**i**), branched-chain (**j**) acylcarnitines, muscle 3-hydroxybutyrate (**k**) and acetoacetate (**l**) and muscle acetyl-CoA (**m**). *TG* triglycerides, *HCC* hydroxylated acylcarnitines, *MCC* medium-chain acylcarnitines, *LCC* long-chain acylcarnitines, *unsaturated C* unsaturated acylcarnitines, *SCC* short-chain acylcarnitines, *DCC* dicarboxylic acylcarnitines, *BCC* branched-chain acylcarnitines, *3HB* 3-hydroxybutyrate, *AcAc* acetoacetate, *CoA* coenzyme A, *rel.* relative, *expr.* expression, *a.u.* arbitrary unit. The interquartile range of HC mice is shown in gray and asterisks above boxplots denote comparisons with HC mice. Statistical significance is shown by */**/***/****: *p* < 0.05/0.01/0.001/0.0001. Illustrations were created with BioRender.com

Muscle TG content and gene expression levels of enzymes involved in cellular (*Cd36*, *Fabp3*) and intramitochondrial (*Cpt1b*) lipid transport, and β-oxidation (*Acadl, Hadha*) were similarly increased in both lipid groups in comparison with the TPN group (Fig. 3a and Figure S5). Plasma TG appeared to accumulate mostly in the LCT group (Fig. 3b), despite both the LCT and gLCT groups receiving equal LCT doses. Cellular lipid handling appeared to be different among groups as the ratio of plasma acylcarnitines to free carnitine, a marker of mitochondrial lipid congestion, was increased in both lipids groups as compared to the TPN-treated mice with the highest increment in the LCT group (Fig. 3c) [22]. This step-wise increase in the gLCT and LCT groups in plasma acylcarnitines was especially pronounced for the hydroxylated, medium- and long-chain, and unsaturated acylcarnitines, byproducts of lipid oxidation (Fig. 3d–g). Conversely, the short-chain acylcarnitines were equally raised in both lipid groups, the dicarboxylic acylcarnitines were increased only in the LCT group, while the branched-chain acylcarnitines, markers of protein catabolism, were not different among the septic mice (Fig. 3h–j).

Plasma 3HB concentrations correlated closely with muscle 3HB levels in the LCT and gLCT groups (Spearman correlation: 0.80, *p* < 0.001; 0.89, *p* < 0.001, resp.). Muscle 3HB and AcAc were progressively higher in the gLCT and LCT group relative to the TPN group (Fig. 3k, l). In the LCT group, 3HB and AcAc correlated strongly (Spearman correlation = 0.81, *p* < 0.001), which was not the case for the gLCT group (Spearman correlation = 0.17, *p* = 0.5). Despite the increased lipid and ketone body availability in the LCT and gLCT groups, muscle acetyl-CoA levels were lower than in the TPN group (Fig. 3m). Expression of ketolytic enzymes (*Oxct1* and *Bdh1*) were not different among these groups (Figure S5). Additionally, gene expression levels of ketone body downstream signaling pathways were overall not differentially affected among the septic mice (Figure S5).

### Supplemental glucose further enhanced hepatic lipid cycling and oxidation but suppressed ketogenesis during LCT infusion

**Fig. 4:**
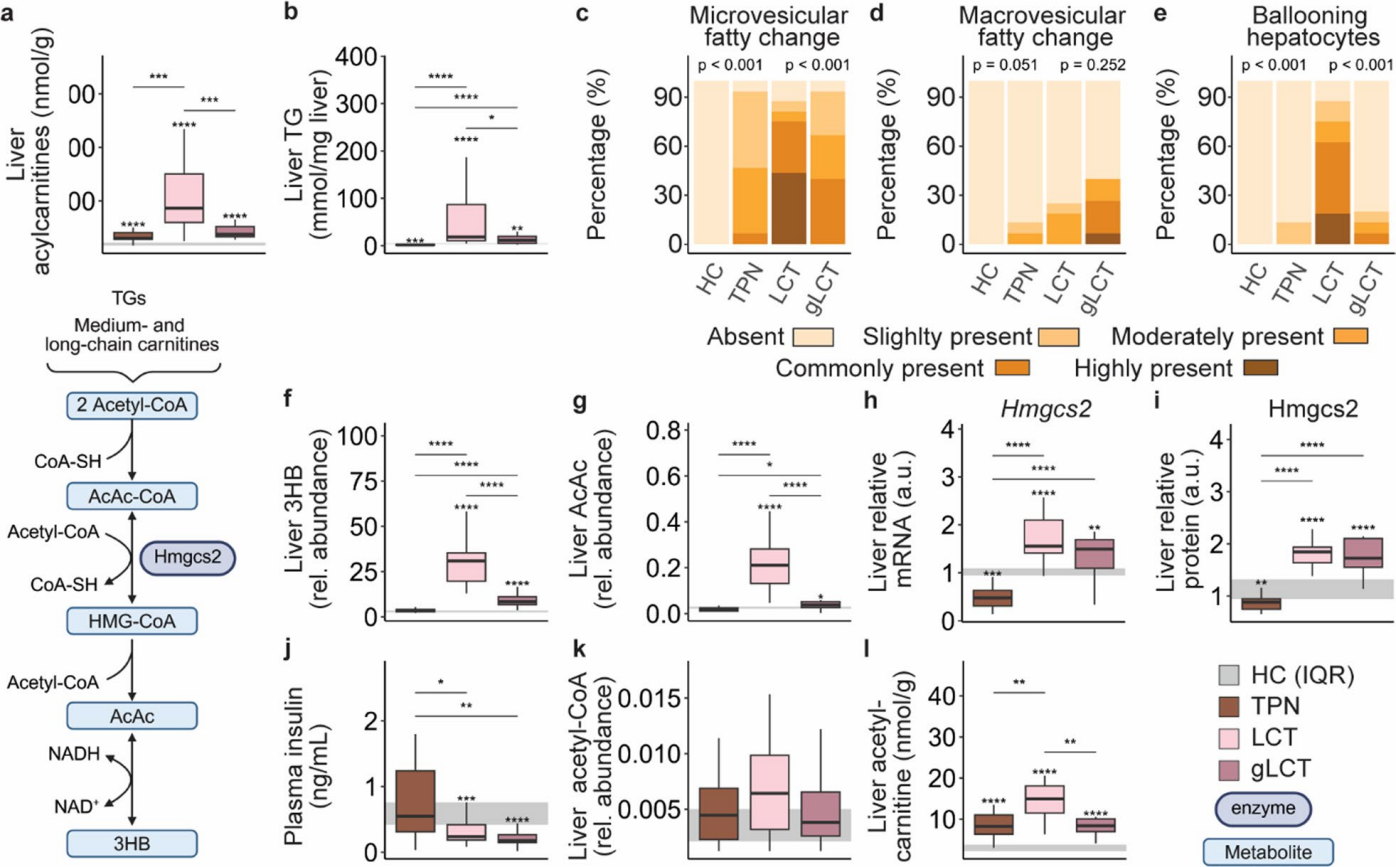
impact of glucose supplemented LCT emulsion vs. a pure LCT emulsion on hepatic acylcarnitines (**a**) and hepatic triglycerides (**b**), histological assessment of microvesicular (**c**) and macrovesicular (**d**) fatty change, and the presence of ballooning hepatocytes (**e**), hepatic 3-hydroxybutyrate (**f**) and acetoacetate (**g**), hepatic relative mRNA (**h**) and protein (**i**) expression of Hmgcs2, plasma insulin (**n**), and liver acetyl-CoA (**o**) and acetylcarnitines (**p**). *TG* triglycerides, *MC* medium-chain, *LC* long-chain, *3HB* 3-hydroxybutyrate, *AcAc* acetoacetate, *a.u.* arbitrary unit, *expr.* expression, *CoA* coenzyme A, *AcAc-CoA* acetoacetyl- coenzyme A, *HMG-CoA* Hydroxymethylglutaryl- coenzyme A. The interquartile range of HC mice is shown in gray and asterisks above boxplots denote comparisons with HC mice. Statistical significance is shown by */**/***/****: p < 0.05/0.01/0.001/0.0001. For histological analyses, left-sided p-values denoted overall statistical comparison assessed by the Kruskal–Wallis test among all groups and right-sided p-values among the septic mice. Illustrations were created with BioRender.com

In line with plasma concentrations, also hepatic acylcarnitine species and TG content were higher in the LCT group relative to the TPN group, and hepatic TG were also higher in the gLCT group as compared to the TPN group (Fig. 4a, b). Only in the LCT group, this hepatic fatty change was accompanied by ballooning hepatocytes (Fig. 4c–e), but overall necrosis and hyperinflammation were limited (Figure S6). Hepatic 3HB and AcAc profiles followed plasma and muscle levels, with the highest concentration during LCT administration and more modest increases in the gLCT group (Fig. 4f, g). Hepatic gene and protein expression of Hmgcs2, the rate-limiting enzyme of ketogenesis was upregulated to the same extent in both LCT groups (Fig. 4h, i and Figure S7). Plasma insulin concentrations decreased during LCT infusion, irrespective of the glucose load (Fig. 4j). Acetyl-CoA hepatic tissue levels, the primary substrate for ketogenesis, were also not different among groups, yet, hepatic acetyl-carnitine was raised in the LCT group (Fig. 4k, l).

### MCT- and LCT-rich infusion with supplemental glucose induced similar mild ketosis and did not further affect muscle weakness

**Fig. 5:**
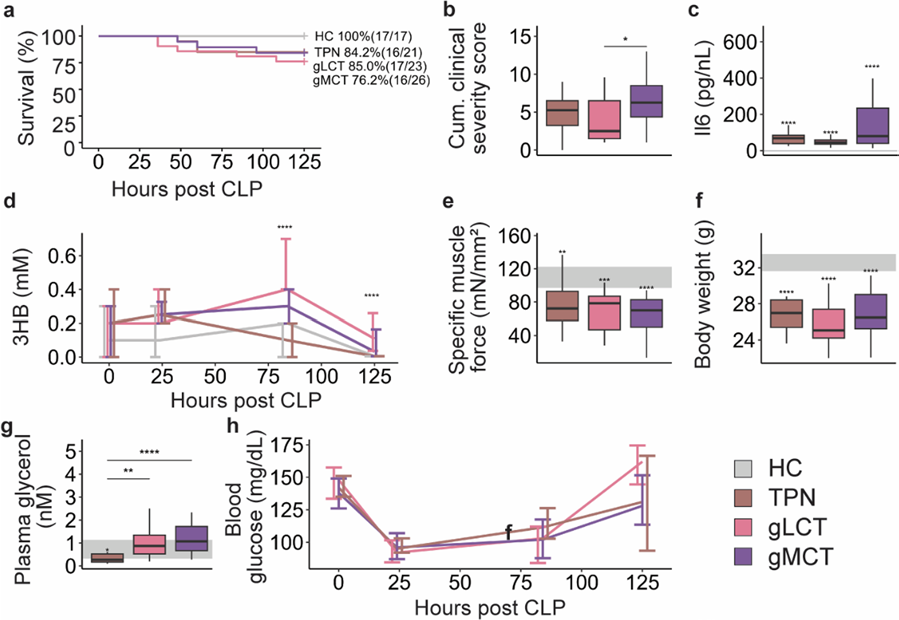
impact of LCT emulsion vs. a mixed MCT/LCT-emulsion, supplemented with glucose, on survival after 5 days of sepsis (**a**), severity of illness (**b**), plasma Il6 concentrations (**c**), ketosis (**d**), specific muscle force (**e**), total body weight (**f**), plasma glycerol (**g**), and blood glucose (**h**). From the 77 animals included in the experiment, 66 animals survived until the end of the study period: healthy control group n = 17/17; sepsis TPN group n = 16/19; sepsis gLCT group n = 17/20; sepsis gMCT group n = 16/21. *CLP* cecal ligation and puncture, *cum.* cumulative, *Il6* interleukin-6, *3HB* 3-hydroxybutyrate. The interquartile range of HC mice is shown in gray and asterisks above boxplots denote comparisons with HC mice. Statistical significance is shown by */**/***/****: *p* < 0.05/0.01/0.001/0.0001. In the line plot, overall statistical difference was assessed by the Kruskal–Wallis test for each time point (0 h, 25 h, 84 h and 125 h)

As the addition of a small amount of glucose to ketogenic formula appeared to be a facilitator of metabolic integrity, but also highly ketogenesis-inhibiting, we performed a second experiment in which we administered an MCT-rich feed to maximize the hepatic ketogenic capacity. Over the course of the experiment, survival was not different among the septic mice (*p* = 0.7) (Fig. 5a). Cumulative clinical severity scores were higher in the gMCT group as compared to the gLCT group, and also Il6 plasma concentrations tended to be higher in this group (*p* = 0.07) (Fig. 5b, c). Unexpectedly, plasma 3HB concentrations were only marginally elevated in the gMCT group and even tended to be lower than in the gLCT group (Fig. 5d). Muscle force was not differentially affected by the nutritional intervention in any of the septic groups (Fig. 5e), nor were histological signs of inflammation, necrosis, fibrosis and fiber shape in the muscle (Figure S8). Total body weight was not affected by the intervention (Fig. 5f). Although MCT emulsions contain proportionally more glycerol than LCT emulsions, glycerol plasma concentrations were similar between the lipid groups, while blood glucose levels appeared lower in the gMCT than the gLCT group (Fig. 5g, h).

### A mixed MCT/LCT-infusion did not enhance lipid oxidation as compared to a LCT-rich emulsion

**Fig. 6:**
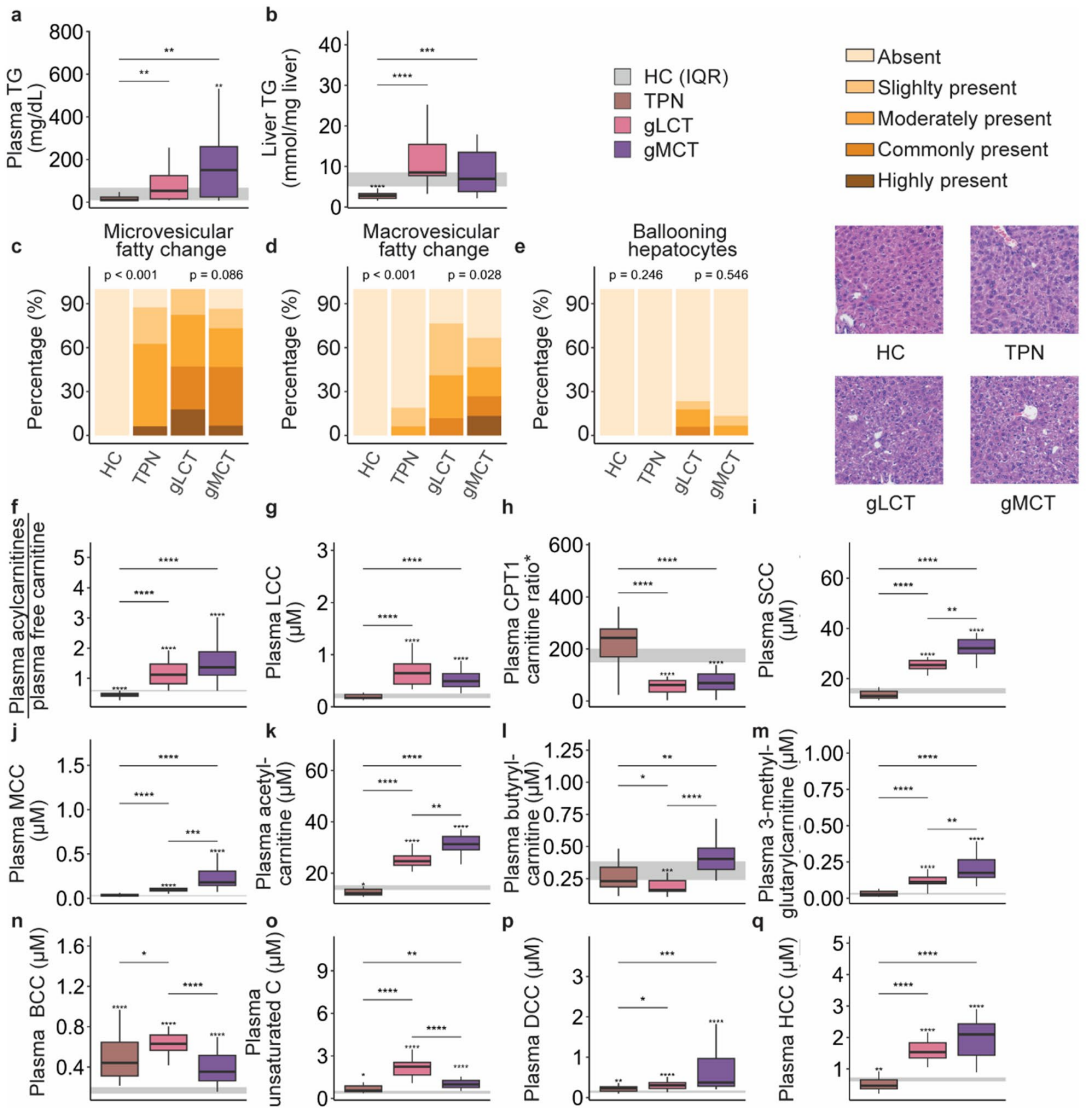
impact of an LCT emulsion vs. a mixed MCT/LCT-emulsion, supplemented with glucose on circulating TGs and acylcarnitines and hepatic fatty change. Plasma TG (**a**), hepatic TG content (**b**), histological assessment of microvesicular (**c**) and macrovesicular (**d**) fatty change, and ballooning hepatocytes (**e**) and representative histology images presented per group, plasma acylcarnitine to free carnitine ratio (**f**), plasma long-chain carnitines (**g**) plasma CPT1 carnitine ratio (*ratio of plasma free carnitine to the sum of palmitoylcarnitine and stearoylcarnitine (**h**), plasma short-chain carnitines (**i**), plasma medium-chain carnitines (**j**), plasma acetylcarnitine (**k**), plasma butyrylcarnitine (**l**), plasma 3-methylglutarylcarnitine (**m**), plasma branched-chain (**n**), unsaturated (**o**), dicarboxylated (**p**) and hydroxylated (**q**) acylcarnitines. *TG* triglycerides, *LC* long-chain, *CPT I* carnitine palmitoyltransferase 1, *LCC* long-chain acylcarnitines, *SCC* short-chain acylcarnitines, *MCC* medium-chain acylcarnitines, *HCC* hydroxylated acylcarnitines, *MCC* medium-chain acylcarnitines, *LCC* long-chain acylcarnitines, *unsaturated C* unsaturated acylcarnitines, *SCC* short-chain acylcarnitines, *DCC* dicarboxylic acylcarnitines, *BCC* branched-chain acylcarnitines. The interquartile range of HC mice is shown in gray and asterisks above boxplots denote comparisons with HC mice. Statistical significance is shown by */**/***/****: *p* < 0.05/0.01/0.001/0.0001. For histological analyses, left-sided p-values denoted overall statistical comparison assessed by the Kruskal–Wallis test among all groups and right-sided p-values among the septic mice. Illustrations were created with BioRender.com

Circulating and hepatic TG concentrations were similarly increased in the gLCT and gMCT groups relative to the TPN group (Fig. 6a, b), with similar histological micro- and macrovesicular fatty changes in hepatocytes (Fig. 6c–e). The ratio of plasma acylcarnitines to free carnitine and the plasma long-chain carnitine levels were also equally increased in both lipid groups relative to the TPN group, despite a lower LCT infusion rate in the gMCT group (Fig. 6f, g). The plasma ratio of free carnitine to the sum of palmitoylcarnitine and stearoylcarnitine, an indication of the functionality of the CPT1 enzyme, was comparably decreased in both lipid emulsion groups (Fig. 6h). The plasma ratio of free carnitine to the sum of palmitoylcarnitine and stearoylcarnitine, an indication of the functionality of the CPT1 enzyme, was comparably decreased in both lipid emulsion groups (Fig. 6h). By contrast, short- and medium-chain acylcarnitines were higher in the gMCT group than the gLCT group (Fig. 6i, j). Among the short-chain acylcarnitines, plasma acetyl- and butyryl-carnitine, downstream products of β-oxidation and peroxisomal oxidation, were markedly elevated in the gMCT group as compared to the gLCT group as was 3-methylglutarylcarnitine, a potential biomarker of mitochondrial dysfunction (Fig. 6k–m) [23, 24]. By contrast, plasma branched-chain acylcarnitines and unsaturated acylcarnitines were lower in the gMCT group as compared to the gLCT group (Fig. 6n, o), and the dicarboxylated and hydroxylated acylcarnitines were similar among these groups (Fig. 6p, q).

### A mixed MCT/LCT-emulsion with supplemental glucose appeared less rather than more ketogenic than LCT infusion with glucose

**Fig. 7:**
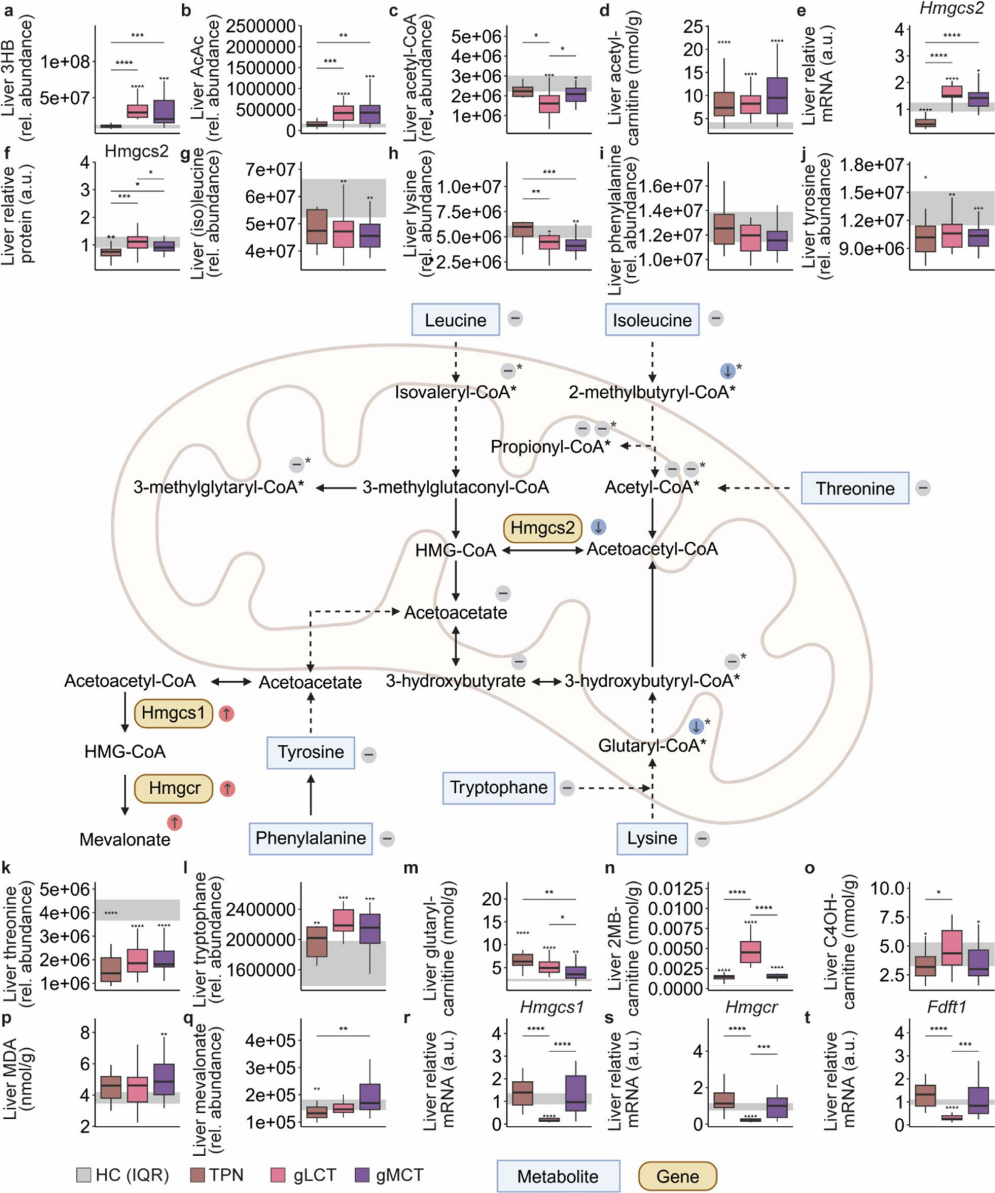
impact of an LCT emulsion vs. a mixed MCT/LCT-emulsion, supplemented with glucose on hepatic ketogenesis. Hepatic 3-hydoxybutyrate (**a**) and acetoacetate (**b**) content, liver acetyl-CoA (**c**), acetylcarnitine (**d**), and relative mRNA (**e**) and protein (**f**) expression Hmgcs2. Hepatic levels of ketogenic amino acids: (iso)leucine (**g**), lysine (**h**), phenylalanine (**i**), tyrosine (**j**), threonine (**k**) and tryptophane (**l**). Hepatic concentrations of glutarylcarnitine (**m**), 2-methylbytyrylcarnitine (**n**), 3-hydroxybutyrylcarnitine (**o**), hepatic MDA content (**p**), hepatic mevalonate content (**q**) and relative mRNA expression of Hmgcs1 (**r**), Hmgcr (**s**), and Fdft1 (**t**). *3HB* 3-hydroxybutyrate, *AcAc* acetoacetate, *CoA* coenzyme A, *AcAc-CoA* acetoacetyl- coenzyme A, *HMG-CoA* Hydroxymethylglutaryl- coenzyme A, *a.u.* arbitrary unit, *C4OH carnitine* 3-hydoxybutyrylcarnitine, *2 MB* 2-methylbutyryl, *MDA* malondialdehyde. Colour coded comparisons in the figure denote hepatic metabolite or relative mRNA expression levels of gMCT group relative to the gLCT group, * refer to the carnitine derivate. The interquartile range of HC mice is shown in gray and asterisks above boxplots denote comparisons with HC mice. Statistical significance is shown by */**/***/****: *p* < 0.05/0.01/0.001/0.0001. For histological analyses, left-sided p-values denoted overall statistical comparison assessed by the Kruskal–Wallis test among all groups and right-sided p-values among the septic mice. Illustrations were created with BioRender.com

To explore the unexpectedly low plasma ketosis, hepatic ketogenic pathways were further explored. In both the gLCT and gMCT groups, plasma 3HB concentrations correlated closely with hepatic 3HB concentrations (Spearman correlation > 0.87, *p* < 0.001), but overall relative abundances tended to be lower in the gMCT group than in the gLCT group (*p* = 0.053) (Fig. 7a). Hepatic acetoacetate levels did not differ among the lipid groups (Fig. 7b). The initial metabolites in ketogenesis, acetyl-CoA and by extension acetyl-carnitine, were mostly similar among the lipids groups (Fig. 7c, d). Gene and protein expression levels of Hmgcs2 was increased in both lipids groups as compared to the TPN group, but to a lesser extent in the gMCT group (Fig. 7e, f and Figure S9). Hepatic levels of ketogenic amino acids ((iso)leucine, lysine, phenylalanine, tyrosine, threonine and tryptophane) were comparable between the lipid groups, but carnitine intermediates including glutaryl-carnitine (C5DC) and 2-methylbutyryl-carnitine of the lysine and isoleucine degradation pathways, respectively, were lower (Fig. 7g–n). Liver 3-hydroxybutyrylcarnitine also tended to be lower in the gMCT group than in the gLCT group (*p* = 0.074) (Fig. 7o). Liver MDA concentrations, a marker of oxidative stress, were comparable between groups (Fig. 7p). Only in the gMCT group, hepatic AcAc concentrations correlated closely with hepatic mevalonic acid concentrations (Spearman correlation = 0.76, *p* < 0.001), which were also globally increased in this group as compared to the TPN group (Fig. 7q). Hepatic mRNA expression of markers of cholesterol synthesis (Hmgcs1, Hmgcr and Fdft1) was markedly lower in the gLCT than the gMCT group (Fig. 7r–t).

## Discussion

This study explored the ketogenic capacity of different lipid-rich PNs in relation to muscle weakness in septic mice. Pure LCT infusion induced stable ketosis but also exacerbated sepsis-induced muscle weakness. Supplemental glucose prevented this decline in muscle force, but also suppressed ketosis, and did not improve muscle function beyond standard PN. Unexpectedly, an MCT-enriched mixture was also ineffective at enhancing ketogenesis or muscle strength.

Pure LCT infusion resulted in a specific metabolic phenotype marked by ketosis, altered muscle glycolytic and TCA metabolites, and an accumulation of plasma acylcarnitines and TGs. The latter metabolic findings suggested a mismatch between lipid supply and oxidative capacity [22, 25]. Remarkably, muscle mass and structure did decline further, and markers of protein catabolism were not markedly increased, suggesting that the exacerbation in muscle weakness likely stemmed from altered metabolic substrate delivery and oxidation [26]. Interestingly, supplemental glucose reversed this exacerbation in muscle weakness and partly normalized most of the changes in glycolytic and TCA metabolites, especially α-ketoglutarate, a pivotal anaplerotic and anabolic metabolite [27]. Moreover, lipid oxidation appeared improved as circulating acylcarnitines were lower, while tissue lipid disposition was not increased [22, 25]. Likewise, previous studies supported improved LCT oxidation in the presence of glucose or its derivatives [22, 28]. Indeed, providing a pure LCT emulsion may have contributed to a β-oxidative bottleneck with sequestration of CoA by long-chain fatty acyls and redox imbalance [22, 29], as observed outside the context of critical illness [22, 29]. Nonetheless, carbohydrate-free ketogenic diets have shown beneficial effects on muscle integrity and function in mice, though typically with added amino acids and in non-septic models [30–33]. The lack of amino acids in the LCT mixture may have exacerbated a relative amino acid-starvation with subsequent impairment of the TCA cycle, further preventing anabolic processes and disrupting the redox balance. However, in critically ill patients the early provision of proteins was demonstrated to worsen muscle weakness and slow down recovery [34, 35].

While supplemental glucose at least partly resolved the global metabolic integrity, it also reduced plasma 3HB concentrations nearly sixfold compared to LCT alone, which appeared to occur mostly independent of insulin and Hmgcs2. A prior study in high-fat-fed mice found a correlation between ketosis and hepatic acetyl-carnitine levels, which were lower in the gLCT group [36, 37]. As other metabolic processes (e.g., the TCA cycle) appeared to be stimulated by supplemental glucose treatment, it may be hypothesized that increased flux through these pathways reduced acetate availability. Remarkably, tracer studies in patients with non-alcoholic fatty liver disease similarly showed acetyl-CoA diverted to the TCA cycle and gluconeogenesis, even despite excess hepatic fatty-acyl groups [38]. Nevertheless, the modest ketosis in the gLCT study could not further improve muscle force, even though prior studies revealed such an effect during ageing-induced and sepsis-induced muscle weakness [6, 39, 40]. The observed ketosis may have simply been too low to activate signaling pathways [6, 33, 41]. A threshold of 0.5 mM is often used to define clinically relevant ketosis, and some authors suggest that 3HB concentrations should increase to > 1–2 mM in order to be effective [6, 42]. Alternatively, glucose may have altered muscle ketone utilization, as muscle 3HB and AcAc correlated only in the LCT group, or may have been insufficient to resolve the potential LCT β-oxidative bottleneck or TCA cycle impairments.

To improve the postulated β-oxidative bottleneck and maximize the ketogenic potential, we tested an MCT-enriched feed [15, 22, 43]. However, this mixture of glucose, MCTs and LCTs did not alleviate sepsis-induced muscle-weakness compared to TPN. Our findings did not support an enhanced oxidation of MCT as plasma long-chain acylcarnitines were equally increased in the gLCT and gMCT groups, despite a lower LCT dose in the gMCT group [25]. Moreover, plasma medium-chain acylcarnitines were higher in the gMCT than in the gLCT group, as were specific acylcarnitines related to β-oxidation, peroxisomal oxidation and mitochondrial dysfunction, suggesting excess non-oxidized circulating acylgroups [25]. The relative contribution of the carnitine shuttle remained uncertain, as the CPT1 acylcarnitine ratio [44] was similar among groups. Nevertheless, this ratio is not validated in the context of critical illness, rendering its interpretation difficult. Furthermore, some studies observed no intramitochondrial MCT transport independent of carnitine in muscle tissue [45, 46].

Surprisingly, our findings did not support the proposed ketogenic effectiveness of MCTs. Compared to the gLCT group, plasma and liver 3HB were lower in the gMCT group, and hepatic Hmgcs2 [47] was relatively downregulated. Although hepatic acetyl-carnitine content was similar, the relative contribution of ketogenic amino acids to ketogenesis may have been lower, as plasma branched-chain and hepatic acylcarnitines of ketogenic amino acid degradation (leucine and lysine) [48] were lower in the gMCT group. In a murine model of cardiac fibrosis, MCT/LCT diets also reduced ketone levels and Hmgcs2 expression compared to LCT-only diets, possibly due to increased oxidative stress during MCT-rich feeds [49]. In the current study, liver MDA levels, a marker of oxidative stress, also tended to be higher in gMCT group. Alternatively, hepatic ketone bodies may have been redirected to other metabolic pathways as hepatic acetoacetate correlated closely with mevalonic acid, a cholesterol precursor, only in the gMCT group. Interestingly, conversion of ketone bodies to cholesterol has been described in both healthy and septic mice [50, 51], for which disturbances in redox potential or a shortage of downstream mevalonate metabolites such as ubiquinone might be contributory factors [50, 52].

Overall, the ketogenic feeds tested were ineffective at inducing ketosis or improving muscle function. In non-critically ill models, ketogenic diets show inconsistent effects on muscle regeneration and performance [33], with some studies reporting muscle benefits, others only an improvement in mitochondrial integrity, or even harm [30, 53–55]. These discrepancies likely reflect differences in metabolic context. In our study, increased lipid load also led to hepatic steatosis. The risk–benefit profile of ketogenic feeds appeared unfavorable under our tested conditions. Parenteral ketone body administration may offer a safer alternative to mimic fasting benefits without steatotic risks. Indeed, exogeneous ketones have been shown to attenuate muscle weakness and promote skeletal muscle regeneration, and may pose a valuable direction for future research [6, 7, 41].

This study has several limitations. First, we did not include sham-operated mice and did not administer antibiotics or pain medication to healthy pair-fed mice, as we did not aim to study the separate impact of sepsis, but rather the complete phenotype of critical illness caused by the complex interplay of disease and ICU treatments. Second, although similar interventions were tested in two experiments, variability in ketosis and survival may reflect inter-animal or seasonal variability [56]. Third, the role of ketogenic amino acids and redox changes was only partially assessed and tissue analyses were based on homogenates limiting insight into subcellular compartmentalization. Moreover, metabolic data were cross-sectional, lacking pathway flux information. Lastly, we tested specific ketogenic diets with fixed macronutrient ratio’s, other compositions may yield different results. Nonetheless, our findings highlight the challenge of safely enhancing ketogenesis via PN in sepsis. The therapeutic window for effective ketogenesis while preserving metabolic integrity is likely narrow and sensitive to interindividual variation.

To conclude, a pure LCT nutrition induced stable ketosis, but exacerbated muscle weakness. Supplemental glucose reversed this decline but suppressed ketogenesis and did not improve muscle function beyond standard PN. A mixed MCT/LCT-nutrition with glucose failed to promote ketosis or muscle strength. Exogenous ketone supplementation may offer a safer and more effective strategy to counteract sepsis-induced muscle weakness, requiring further investigation.

## Supplementary Information

Supplementary material 1.

## Data Availability

All datasets generated during and/or analyzed during the current study are available on the research data repository of the KU Leuven and accessible upon request to the PI.

## Abbreviations

AcAc: Acetoacetate
C5DC: Glutaryl-carnitine
C4OH: Hydroxybutyrylcarntine
CLP: Caecal ligation and puncture
CoA: Coenzyme A
DHAP: Dihydroxyacetone phosphate
EDL: M. extensor digitorum longus
FFA: Free fatty acids
GADP: Glyceraldehyde-3-phosphate
H&E: Hematoxylin and eosin
HC: Healthy control
ICUAW: Intensive care unit-acquired weakness
LC–MS-MS: Liquid chromatography with tandem mass spectrometry
LCT: Long-chain triglyceride
MCT: Medium-chain triglyceride
MDA: Malondialdehyde
mRNA: Messenger RNA
PEP: Phosphoenolpyruvate
TCA: Tricarboxylic acid cycle
TG: Triglycerides
TPN: Total parenteral nutrition
3HB: 3-Beta-hydroxybutyrate
Acadl: Acyl-Coenzyme A dehydrogenase, long-chain
Acox1: Acyl-Coenzyme A oxidase 1, palmitoyl
Atgl: Adipose triglyceride lipase
Bdh1: 3-Hydroxybutyrate dehydrogenase, type 1
Cd36: CD36 molecule
Cpt1a: Carnitine palmitoyl transferase 1a
Cpt1b: Carnitine palmitoyl transferase 1b
Cs: Citrate synthase
Fabp3: Fatty acid binding protein 3, muscle and heart
Hadha: Hydroxyacyl-CoA dehydrogenase trifunctional multienzyme complex subunit alpha
Hk1: Hexokinase 1
Hmgcs2: 3-Hydroxy-3-Methylglutaryl-CoA Synthase 2
Idh2: Isocitrate dehydrogenase 2, mitochondrial
Il6: Interleukin 6
Oxct1: 3-Oxoacid CoA transferase 1
Pck1: Phosphoenolpyruvate carboxykinase 1, cytosolic
Pcx: Pyruvate carboxylase
Pdk4: Pyruvate dehydrogenase kinase, isoenzyme 4
Pfkm: Phosphofructokinase, muscle
Pkm: Pyruvate kinase, muscle
Rn18s: 18S ribosomal RNA
Sdha: Succinate dehydrogenase complex, subunit A, flavoprotein (Fp)
Tnf: Tumor necrosis factor
