# Supplementary material for: The impact of lipid-rich nutrition on ketogenesis and muscle weakness in sepsis
Source: Intensive Care Med Exp. 2026 Feb 12;14:17. doi: 10.1186/s40635-026-00867-8 (PMC12901813; doi:10.1186/s40635-026-00867-8)
Supplement: Supplementary file 1 — Supplementary material 1. [file 40635_2026_867_MOESM1_ESM.pdf]

SUPPLEMENTAL FILE

The impact of lipid-rich nutrition on ketogenesis and muscle weakness in sepsis

Caroline Lauwers[1], Jan Gunst[1], Soraya El Dawy[1], Sarah Derde[1], Lies Pauwels[1], Inge Derese[1], Sarah Vander Perre [1], Greet Van den Berghe[1], Michael P. Casaer[1], Lies Langouche[1]

1 Department of Cellular and Molecular Medicine, Laboratory and Clinical division of Intensive Care Medicine, KU Leuven, Leuven, Belgium.

SUPPLEMENTARY METHODS

Ex vivo muscle force measurements

Ex vivo muscle force was measured in surviving septic mice and healthy control mice in the hindlimb m. extensor digitorum longus (EDL) as the primary outcome. Directly after terminating the experiment, the EDL was suspended in a temperature controlled (30°C) organ bath filled with HEPES-fortified Krebs-Ringer solution (300C-LR Dual-Mode muscle lever, Aurora Scientific, Ontario, Canada). After initializing the muscle to the resting length, the maximal isometric tetanic force was measured as the average force generated by three consecutive tetanic stimuli (180 Hz stimulation frequency, 200ms duration, 0.2 ms pulse width, 2 min rest intervals), and divided by the muscle cross-sectional area to calculate the specific maximal isometric tetanic force.

Whole blood and plasma analyses

Throughout the experiment, a point-of-care tester (StatStrip Xpress 2; Nova Biomedical, Waltham, MA) measured 3-hydroxybutyrate (3HB) concentrations on whole blood drawn from the tail vein at the start of the experiment, on the morning of day 1 (before the initiation of PN) and on the evening of day 3. All other measurements were performed on plasma collected with a cardiac puncture at the end (day 5) of the experiment. Commercial assays were used to assess plasma concentrations of triglycerides (TG, Abcam, Cambridge, UK) and tumor necrosis factor α and Il-6 (R&D Systems, Abingdon, UK), and malondialdehyde (MDA, Abcam, Cambridge, UK). Plasma 3HB concentrations were quantified with an internally developed enzymatic assay with a detection limit of 0.005 mmol/L.[1]

Tissue analyses

Water content, gene expression and metabolite tissue content were assessed on the gastrocnemius muscle and liver tissue. Dry mass and water content was measured by a freeze-drying process (frozen tissue samples were dried for 3 hours on 100 °C). The Qiazol and the

RNeasy mini RNA isolation kit (QIAGEN, Venlo, The Netherlands) was used to isolate RNA, after which DNAse treatment was applied to remove genomic DNA, and RNA was reverse transcribed with the use of random hexamers. Relative gene expression was determined with the 2-ΔΔCt method with 18S ribosomal RNA (*Rn18s*) as housekeeping gene for liver tissue and succinate dehydrogenase complex flavoprotein subunit A (*SDHA*) for muscle tissue by commercial TaqMan® assays (Applied Biosystems, Carlsbad, CA, USA) (Supplementary Table S2). Liver triglycerides were measured (FUJIFILM Wako, Richmond, USA) after hexane extraction. Commercial assays (Abcam, Cambridge, UK) were used to measure muscle glycogen content.

Immunoblotting was conducted for Hmgcs2 on liver tissue with alpha-tubilin as housekeeping protein. Tissue samples were homogenized in a buffer 1% Nonidet P-40; 10% glycerol; 20 mM Tris-HCl (pH = 7.6); 10 mM EDTA; 1x Complete Mini. The protein content was determined with Coomassie Protein Assay Reagent (Pierce Biotechnology Inc.) using a standard curve of BSA. Western blots were performed using commercial 4-20% tris-glycine gels (Biorad, Hercules, CA) and PVDF membranes (Thermo Fisher scientific, Waltham, MA). Secondary horseradish peroxidase-conjugated antibodies were purchased from DakoCytomation (Heverlee, Belgium). Blots were developed with the Western Lightning chemiluminescence reagent Plus kit (Perkin Elmer, Zaventem, Belgium), visualized with the G:BOX Chemi XRQ (SynGene, Cambridge, UK) and analyzed with the SynGene software.

Liver (left lobe) tissue and muscle (m. tibialis) was processed to cross-sectional paraffin sections, stained by hematoxylin and eosin (H&E). Liver sections were scored semi-quantitatively for inflammation, necrosis, and steatosis. Steatosis parameters included the presence of microvascular and macrovascular fatty change, and the presence of ballooning hepatocytes. Muscle sections were assessed semi-quantitatively for inflammation, necrosis, fibrosis and fiber shape. Assessors were blinded to the nutritional allocation.

Metabolomics

Targeted metabolomics of muscle (first study) and liver (first and second study) tissue were performed with a Dionex UltiMate 3000 LC System (Thermo Scientific Bremen, Germany) equipped with a C-18 column (Acquity UPLC -HSS T3 1. 8 µm; 2.1 x 150 mm, Waters) coupled to a Q Exactive Orbitrap mass spectrometer (Thermo Scientific) operating in negative ion mode in collaboration with the metabolomics expertise center (KU Leuven). The Xcalibur software (Thermo Scientific) was used for data collection. For data analysis peak areas were integrated (El-Maven – Polly - Elucidata). Data analysis of metabolomics data was conducted by the MetaboAnalystR package.[2] Data were normalized according to variability in healthy control samples. Joint pathway analysis was conducted to identify pathways of the KEGG mouse database that were significantly altered between septic groups (hypergeometric test), based on metabolites and gene expression profiles that differed significantly among the groups to be compared. Topological importance was determined by betweenness centrality measures. The overall pathway significance was determined by integrating both enrichment and topological results and reported as combined p-values. Pathways were ranked based on these overall p-values, with lower values indicating higher relevance. Correlations were calculated between metabolites by Spearman correlation. Only significant ($p < 0.05$) correlations were reported.

Carnitine profiling

Plasma and liver acylcarnitine profile and free carnitine concentrations were assessed by liquid chromatography with tandem mass spectrometry (LC-MS-MS) in collaboration with the Metabolomics Innovation Centre (Victoria, Canada). After sample preparation, carnitines were analyzed with LC-MRM/MS with (+) ion detection on an Agilent Infinity II 1290 UHPLC system coupled to an Agilent 5495C QQQ mass spectrometer. A binary-solvent mobile phase consisting of a perfluorobutanoic acid buffer and acetonitrile was used for gradient elution. LC-MRM/MS was carried out under optimized separation and detection conditions. Linear regression calibration curves for individual carnitines were constructed using data acquired from the injected

calibration solutions. The concentrations of carnitines detected were calculated by interpolating the calibration curves with data from the injected sample solutions within appropriate concentrations.

Statistical analyses

Median and interquartile ranges were used as data summary measures and represented by boxplots of which the whiskers extend until the furthest point within 1.5 times the interquartile range. After visual inspection of the distribution of the data, the Analysis of Variance (ANOVA) or Student's t-test were applied in case of a Gaussian distribution, or the Kruskal-Wallis test or Wilcoxon signed-rank test if the data was skewed, as appropriate. Kaplan-Meier survival curves were assessed by the log-rank test. Two-sided p-values ≤ 0.05 were considered statistically significant. Statistical analyses were conducted with the R statistical programming language (R Core Team (2023). _R: A Language and Environment for Statistical Computing_. R Foundation for Statistical Computing, Vienna, Austria. <https://www.R-project.org/>., (version 4.5.0)) and the RStudio interface (version 2025.05.0, Boston, MA, USA).

Supplementary Table S1: theoretical macronutrient composition of parenteral nutritional formulas shown as a percentage of the total caloric intake of the TPN group (5.27 kcal/d)

	TPN	LCT	gLCT	gMCT
Carbohydrates	51.0%	0%	10%	10%
Amino acids	16.2%	0%	0%	0%
LCTs	38.2%	90%	90%	45%
MCTs	0%	0%	0%	45%

Abbreviations: TPN: total parenteral nutrition; LCTs: long-chain triglyceride; MCTs: medium-chain triglycerides.

Supplementary Table S2: list of TaqMan gene expression assays

Gene name	TaqMan Assay ID
Acadl	
Bdh1	Mm00558330_m1
Cd36	Mm00432403_m1
Cpt1b	Mm00487191_g1
Fabp3	Mm02342495_m1
Hadha	Mm00805228_m1
Hmgcs2	Mm00550050_m1
Il6	Mm00446190_m1
Myh1	Mm01332489_m1
Myh2	Mm01332564_m1
Myh4	Mm01332541_m1
Myh7	Mm00600555_m1
Oxct1	Mm00499303_m1
Rn18s	Mm03928990_g1
Tnf	Mm00443258_m1
Nlrp3	Mm00840904_m1
Il1b	Mm00434228_m1
SDHA	Mm01352366_m1

SUPPLEMENTARY FIGURES

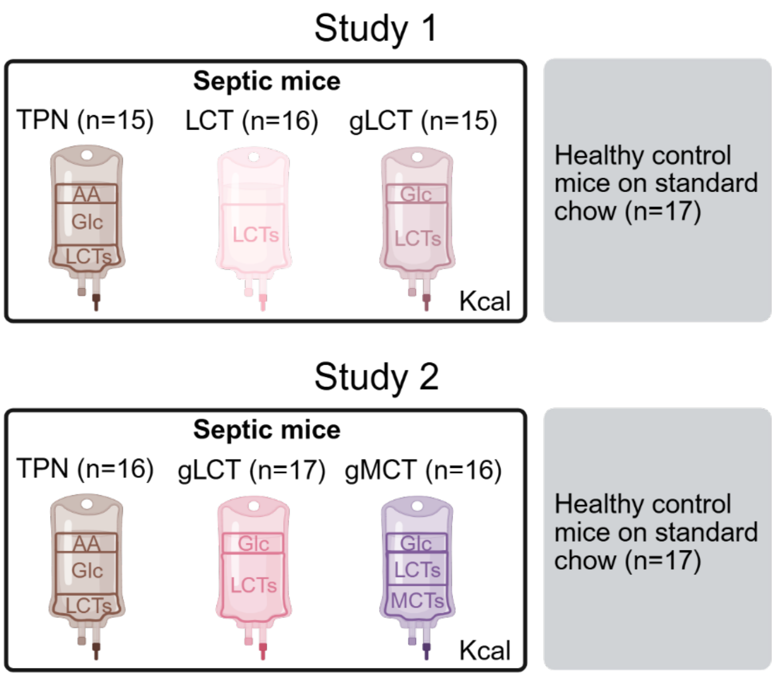

Supplementary figure S1: visual representation of randomized intervention in study 1 and study 2. Abbreviations: AA: amino acids; Glc: glucose; LCTs: long-chain triglyceride; MCTs: medium-chain triglycerides; Kcal: kilocalories.

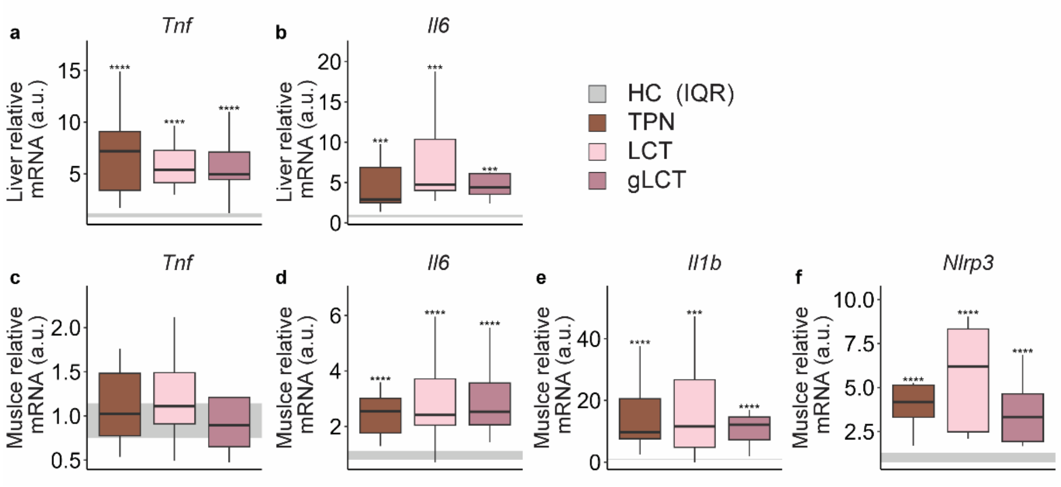

Supplementary Fig. S2: relative mRNA expression of hepatic Tnf (a), il6 (b) and muscle Tnf (c), il6 (d), Il1b (e) and, Nlrp3 (f). Abbreviations: a.u.: arbitrary unit. The interquartile range of HC mice is shown in gray and asterisks above boxplots denote comparisons with HC mice. Statistical significance is shown by */**/***/****: p<0.05/0.01/0.001/0.0001.

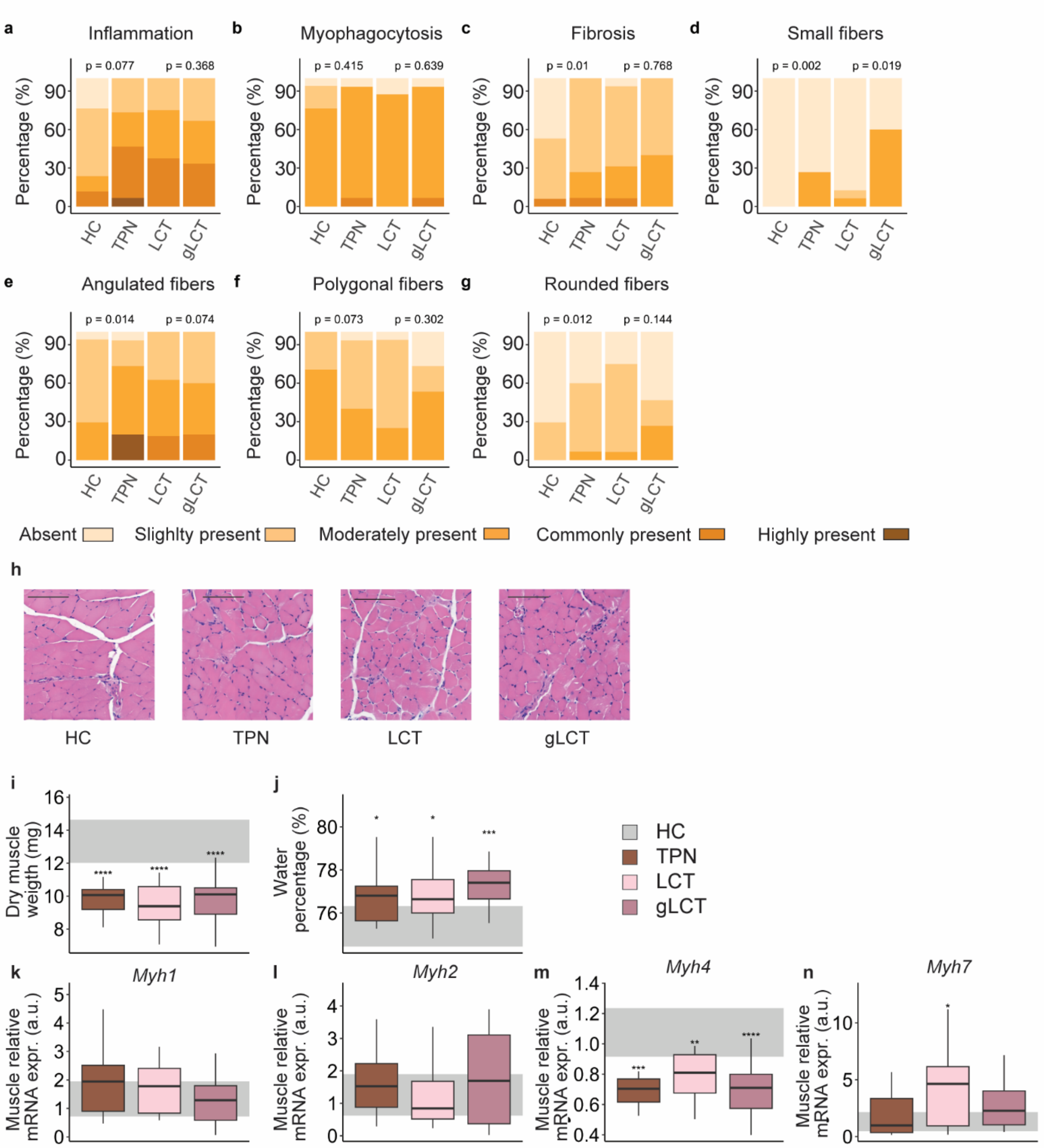

Supplementary Fig. S3: impact of glucose supplemented LCT emulsion vs. a pure LCT emulsion on muscle inflammation (a), myophagocytosis (b), fibrosis (c), and the presence of small fibers (d), angulated (e), polygonal (f) and rounded (g) fibers. Representative images are represented per group (h). Dry muscle weight (i) and the percentage of muscle water (j), and gene expression levels for Myh1 (k), Myh2 (l), Myh4 (m) and Myh7 (n) are displayed per group. Abbreviations: a.u.: arbitrary unit. Left-sided p-values denoted overall statistical comparison assessed by the Kruskal-Wallis test among all groups and right-sided p-values among the septic mice.

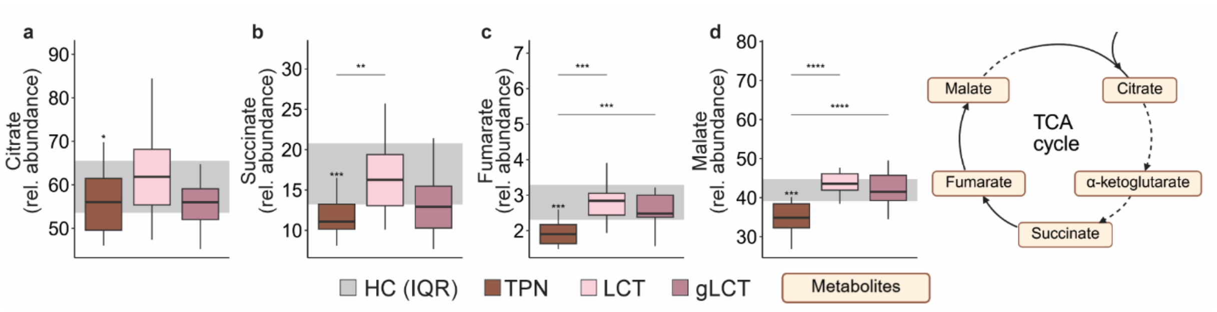

Supplementary Fig. S4: impact of glucose supplemented LCT emulsion vs. a pure LCT emulsion on muscle citrate (a), succinate (b), fumarate (c) and malate (d) levels. Abbreviations: TCA: tricarboxylic acid: CoA: coenzyme A. The interquartile range of HC mice is shown in gray and asterisks above boxplots denote comparisons with HC mice. Statistical significance is shown by */**/***/****: $p<0.05/0.01/0.001/0.0001$. Illustrations were created with BioRender.com.

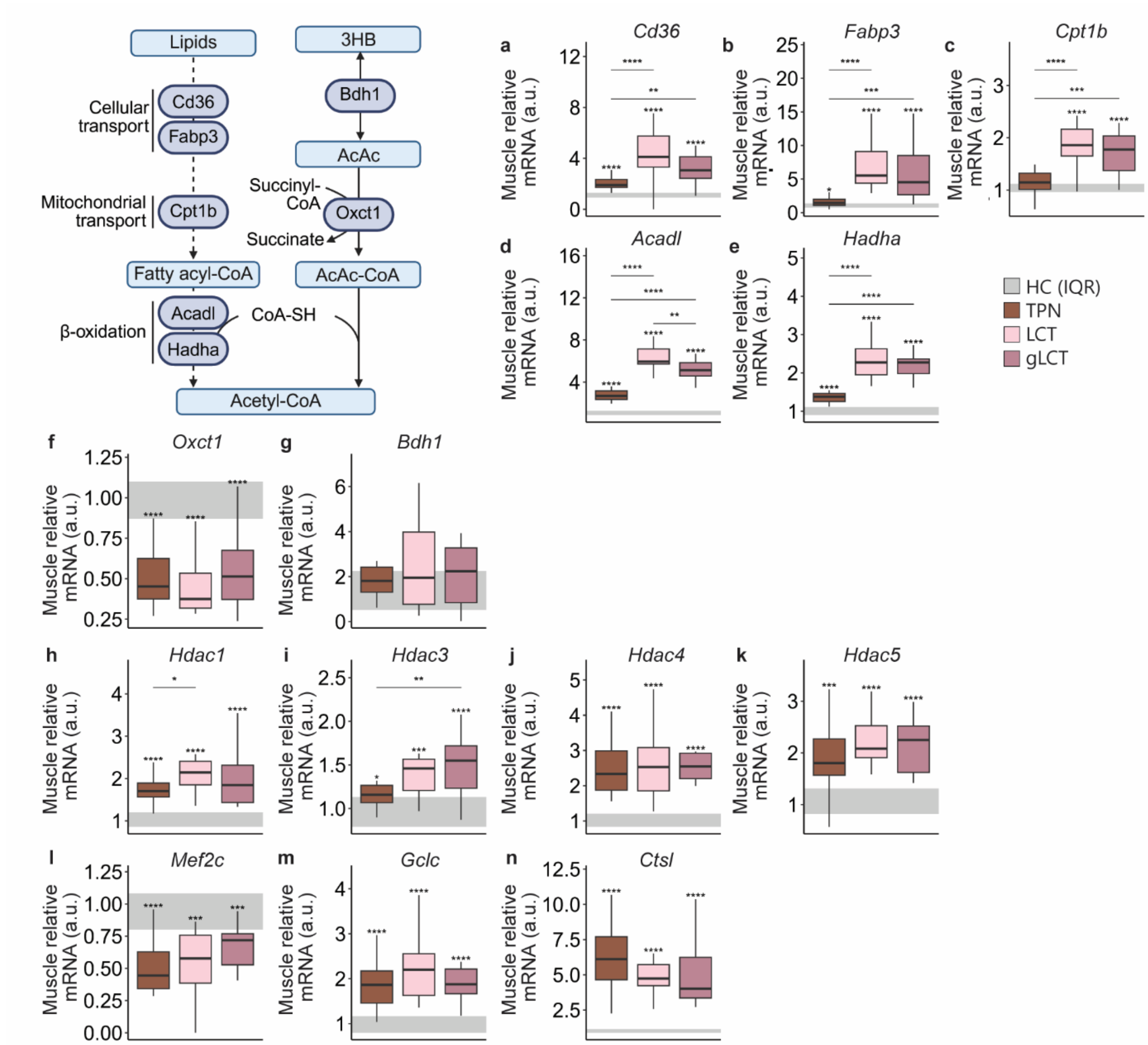

Supplementary Fig. S5: relative mRNA expression of enzymes involved in lipid oxidation, *Cd36* (a), *Fabp3* (b), *Cpt1b* (c), *Acadl* (d), *Hadha* (e); and in ketolysis, *Oxct1* (f) and *Bdh1* (g); and in muscle ketone body signaling pathways: *Hdac1* (a), *Hdac3* (b), *Hdac4* (c), *Hdac5* (d), *Mef2c* (e) *Gclc* (f) *and* Ctsl (g). Abbreviations: 3HB: 3-hydroxybutyrate; AcAc: acetoacetate; CoA: coenzyme A; AcAc-CoA: acetoacetyl- coenzyme A; a.u.: arbitrary unit. The interquartile range of HC mice is shown in gray and asterisks above boxplots denote comparisons with HC mice. Statistical significance is shown by */**/***/****: p<0.05/0.01/0.001/0.0001.

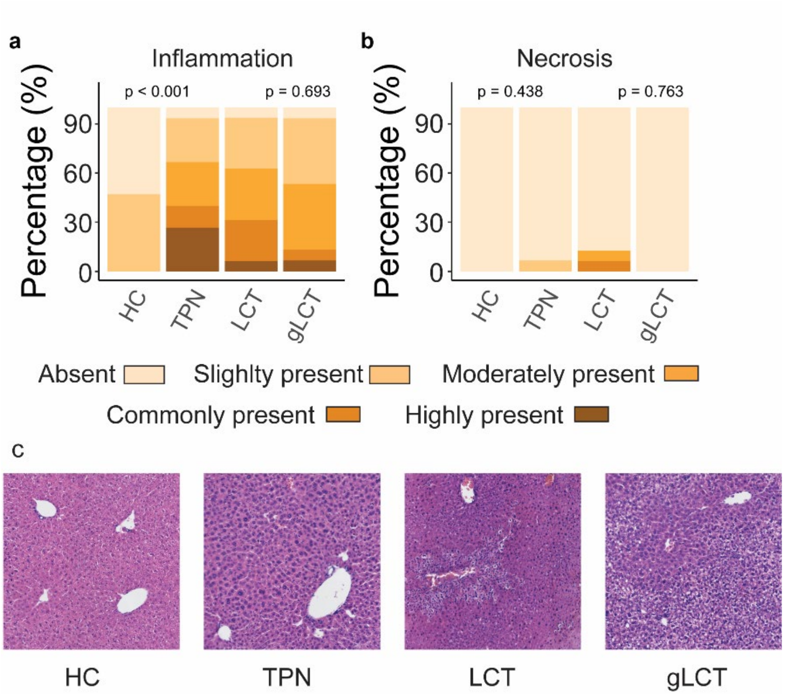

Supplementary Fig. S6: impact of glucose supplemented LCT emulsion vs. a pure LCT emulsion on hepatic inflammation (a) and necrosis (b). Representative images are represented per group (c). Left-sided p-values denoted overall statistical comparison assessed by the Kruskal-Wallis test among all groups and right-sided p-values among the septic mice.

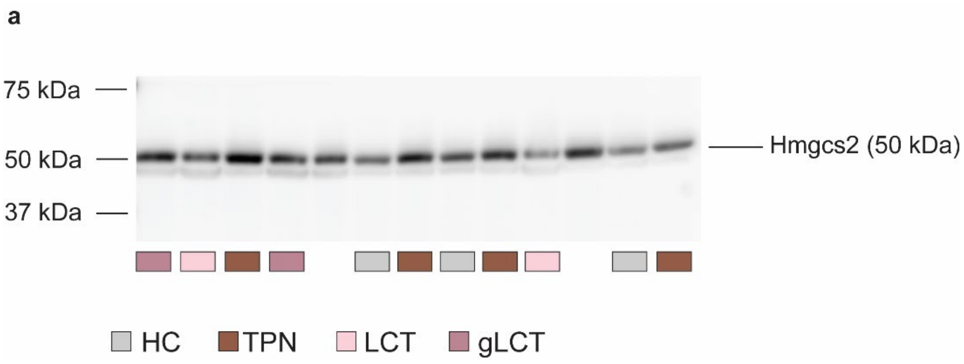

Supplementary Fig. S7: Illustration of Western blots on liver of healthy and critically ill mice for Hmgcs2 – study 1.

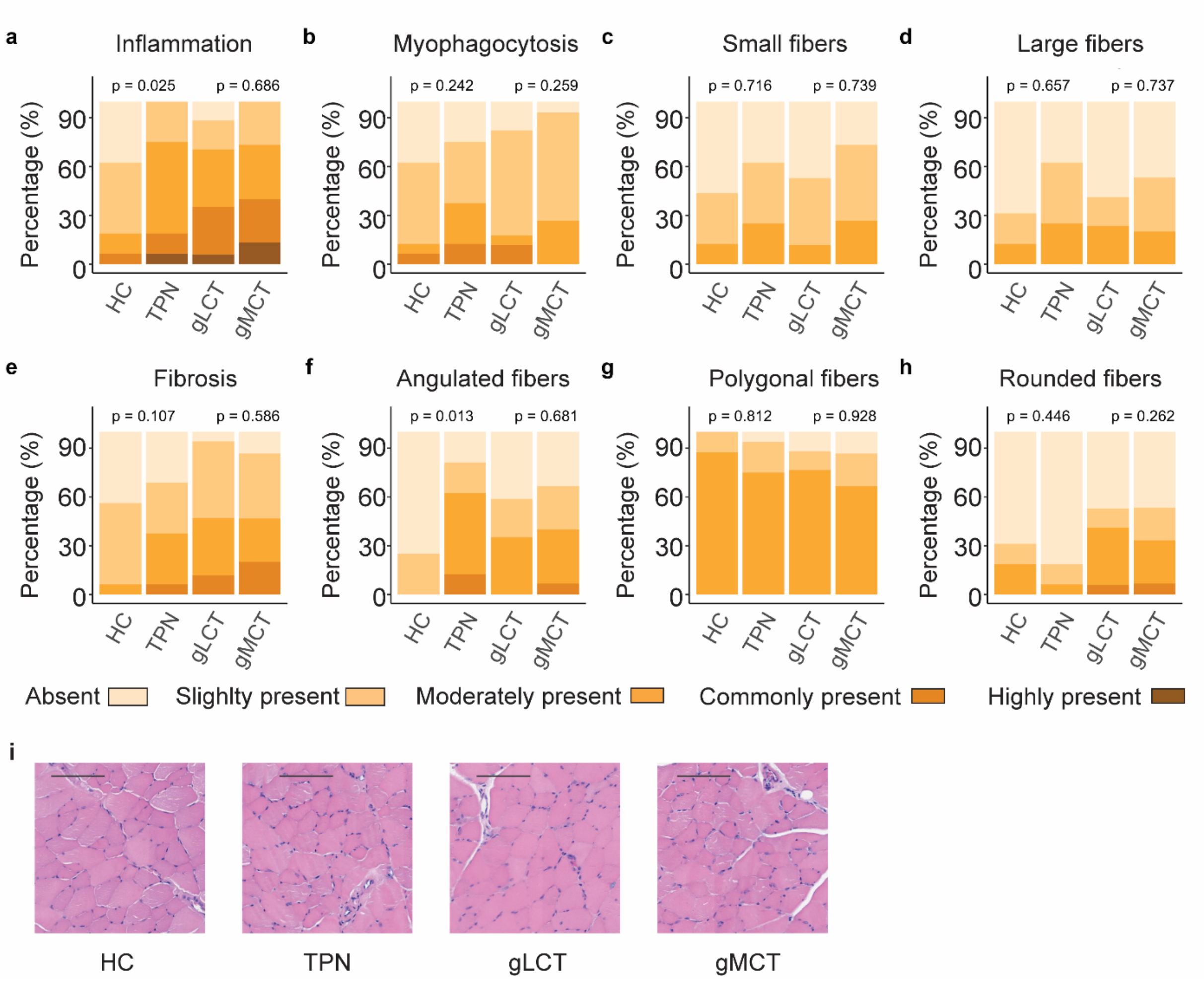

Supplementary Fig. S8: impact of glucose supplemented LCT emulsion vs. a glucose supplemented MCT-rich emulsion on muscle inflammation (a), myophagocytosis (b), and the presence of small (c) and larger (d) fibers, fibrosis (e), and the presence of angulated (f), polygonal (g) and rounded (h) fibers. Representative images are represented per group (i). Left-sided p-values denoted overall statistical comparison assessed by the Kruskal-Wallis test among all groups and right-sided p-values among the septic mice.

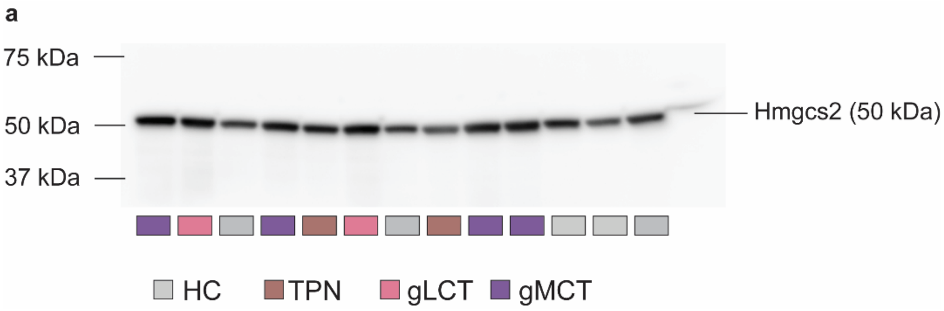

Supplementary Fig. S9: Illustration of Western blots on liver of healthy and critically ill mice for Hmgcs2 in study 2.

REFERENCES

1 De Bruyn, A. *et al.* Effect of withholding early parenteral nutrition in PICU on ketogenesis as potential mediator of its outcome benefit. *Crit Care* **24**, 536 (2020). https://doi.org/10.1186/s13054-020-03256-z

2 Pang, Z. *et al.* MetaboAnalyst 6.0: towards a unified platform for metabolomics data processing, analysis and interpretation. *Nucleic Acids Res* **52**, W398-w406 (2024). https://doi.org/10.1093/nar/gkae253
